# Substance P Inhibits Hyperosmotic Stress-Induced Apoptosis in Corneal Epithelial Cells through the Mechanism of Akt Activation and Reactive Oxygen Species Scavenging via the Neurokinin-1 Receptor

**DOI:** 10.1371/journal.pone.0149865

**Published:** 2016-02-22

**Authors:** Lingling Yang, Wenjie Sui, Yunqiu Li, Xia Qi, Yao Wang, Qingjun Zhou, Hua Gao

**Affiliations:** 1 State Key Laboratory Cultivation Base, Shandong Provincial Key Laboratory of Ophthalmology, Shandong Eye Institute, Shandong Academy of Medical Sciences, Qingdao, China; 2 Shandong Eye Hospital, Shandong Eye Institute, Shandong Academy of Medical Sciences, Qingdao, China; National Cheng Kung University, TAIWAN

## Abstract

Hyperosmolarity has been recognized as an important pathological factor in dry eye leading to ocular discomfort and damage. As one of the major neuropeptides of corneal innervation, substance P (SP) has been shown to possess anti-apoptotic effects in various cells. The aim of this study was to determine the capacity and mechanism of SP against hyperosmotic stress-induced apoptosis in cultured corneal epithelial cells. The cells were exposed to hyperosmotic stress by the addition of high glucose in the presence or absence of SP. The results showed that SP inhibited hyperosmotic stress-induced apoptosis of mouse corneal epithelial cells. Moreover, SP promoted the recovery of phosphorylated Akt level, mitochondrial membrane potential, Ca^2+^ contents, intracellular reactive oxygen species (ROS) and glutathione levels that impaired by hyperosmotic stress. However, the antiapoptotic capacity of SP was partially suppressed by Akt inhibitor or glutathione depleting agent, while the neurokinin-1 (NK-1) receptor antagonist impaired Akt activation and ROS scavenging that promoted by SP addition. In conclusion, SP protects corneal epithelial cells from hyperosmotic stress-induced apoptosis through the mechanism of Akt activation and ROS scavenging via the NK-1 receptor.

## Introduction

Substance P (SP), an 11-amino acid neuropeptide of the tachykinin family, has been shown broadly distributed in the central and peripheral nervous system with multiple functions in both physiological and pathological conditions[1–3]. It mainly functions through the interaction with neurokinin receptors, among which the neurokinin-1 (NK-1) receptor shows a preferential affinity for SP [1, 4, 5]. Recent studies have shown that SP possesses anti-apoptotic effects in a variety of cells, such as kidney epithelial cells [6], colonocytes [7], cerebellar granule cells [8], spiral ganglion neurons [9], dendritic cells [10], tenocytes [11, 12] and even cancer cells [13]. Evidence indicates that SP activates the phosphoinositide 3-kinase-Akt (PI3K-Akt), mitogen-activated protein kinases (MAPK), extracellular signal regulated kinases (ERK) and epidermal growth factor receptor (EGFR) signaling pathways [6, 7, 9, 11, 14], among which the activation of PI3K-Akt plays an important role in its anti-apoptotic activity. Several researches have demonstrated that SP reduces apoptotic response by binding to the NK-1 receptor [7, 9, 11, 13]. However, the downstream mechanism of SP-mediated anti-apoptosis effect remains unknown.

Apoptosis is programmed cell death that mediated by at least the mitochondrial pathway [15] and the cell death receptor pathway [16]. It has been well known that pro-apoptotic Bcl-2 proteins Bcl-2-associated death promoter (Bad) and BCL2-associated X protein (Bax) translocate to mitochondria upon induction of apoptosis. Moreover, excessive accumulation of intracellular reactive oxygen species (ROS) plays an important role in apoptosis induction under both physiologic and pathologic conditions [17–21]. Mitochondria are the main source and target of ROS [19, 20]. ROS triggers the mitochondrial apoptotic pathway by promoting the loss of membrane polarization, the release of cytochrome c from mitochondria and caspase activation [21, 22], while the attenuation of ROS accumulation prevents the apoptosis in endothelial cells [23]. In contrast to cytochrome c, apoptosis-inducing factor (AIF) is another pro-apoptotic mitochondrial protein acts in a caspase-independent fashion [24].

The corneal epithelial layer covers the front of the cornea and acts as a barrier to protect the structures behind the cornea from damages caused by environmental insults and infections [25]. Tear hyperosmolarity is thought to be the central mechanism in the pathogenesis of ocular surface damage from dry eye. Elevated tear osmolarity are often found in some pathologic conditions, such as diabetes mellitus [26–28], dehydration after exercise [29], contact lens wear [30] and inflammation [31]. Although it is hard to measure directly, it has been proposed that the tear film osmolarity over the ocular surface can increase from 450 to 600 mOsm [32–34] compared with 300–310 mOsm in normal eyes. Several studies have shown that hyperosmotic stress can induce apoptosis in corneal epithelial cells [35–38]. The inhibition of apoptosis may be an effective measure to protect cornea from the damage of hyperosmotic stress. In the present study, we established a hyperosmotic stress-induced apoptosis model by high glucose in mouse corneal epithelial cells and explored the protective role and mechanism of substance P from hyperosmotic stress-induced apoptosis. We found that SP inhibits corneal epithelial cells apoptosis induced by hyperosmotic stress in vitro, partially through the activation of Akt signaling and the direct ROS scavenging capacity of SP via NK-1 receptor.

## Materials and Methods

### Corneal epithelial cell culture and treatment

Mouse corneal epithelial cell line (TKE2) was presented from Dr. Tetsuya Kawakita of Keio University (Tokyo, Japan) and cultured in keratinocyte serum-free medium (KSFM, Invitrogen, Carlsbad, CA) [39]. To induce apoptosis caused by hyperosmotic stress, TKE2 cells were incubated overnight in bovine pituitary extract (BPE)-free KSFM and subsequently exposed to hypertonic media (450, 550 and 650 mOsm) achieved by addition of glucose for 12–48 h. To detect the inhibiting effects on hyperosmotic stress-induced apoptosis, 0.1, 1 or 10 μM SP (Calbiochem, San Diego, CA) was pre-incubated 2 h before the treatment of high glucose. In some experiments, TKE2 cells were treated with the NK-1 receptor antagonist (1 μM L-733,060, Tocris, Minneapolis, MN), the AKT inhibitor V (40 μM Triciribine, Calbiochem, San Diego, CA), or glutathione depleting agent L-Buthionine-sulfoximine (100 μM, L-BSO, Sigma-Aldrich, St. Louis, MO) in the presence or absence of substance P.

### Annexin V/PI Assay

The quantification of cell apoptosis was determined using the Annexin V/propidium iodide (PI) apoptosis detection kit according to the manufacturer’s instructions (BD Pharmingen, San Jose, CA). In brief, the collected cells were suspended in binding buffer and incubated with Annexin V-FITC and PI for 15 min at room temperature. The samples were determined by FACScalibur flow cytometry (BD Bioscience) and the data was analyzed using FlowJo software.

### Caspase activity assays

Assays for caspase 3, 8, and 9 were detected according to the manufacturer’s protocol (Beyotime, Haimen, China). Briefly, the cell lysates were incubated with 200 μM Ac-DEVD-AMC (substrate for caspase 3), Ac-IETD-pNA (substrate for caspase 8) or Ac-LEHD-pNA (substrate for caspase 9) at 37°C for 60–120 min. The absorbance of A405 was measured using a MultiMode Microplate Reader (SpectraMax M2, Molecular Devices, Menlo Park, CA). Caspase activities were normalized with the total protein contents.

### Immunofluorescence staining

Cells were fixed by 4% paraformaldehyde for 15 min, permeabilized with 0.1% Triton X-100 for 5 min and blocked with 5% bovine serum albumin (BSA) for 1 h. The samples were stained with primary antibodies against AIF (Santa Cruz Biotechnology, Santa Cruz, CA), p-Akt (pS473, Epitomics, Burlingame, CA), MnSOD, catalase, NQO1, Hmox1, Bad and Bax (abcam, Cambridge, MA) at a concentration of 1:100 overnight at 4°C, and then incubated with fluorescein-conjugated secondary antibodies (Santa Cruz Biotechnology) at 37°C for 30 min. The cells were then counterstained with 4’,6-diamidino-2-phenylindole (DAPI, Sigma-Aldrich). All staining was examined under a confocal laser-scanning microscope.

### Western blot analysis

Total protein was lysed from TKE2 cells in RIPA buffer. Samples were run on 12% SDS-PAGE gels for 2 h at 110 V and then transferred to a PVDF membrane (Millipore, Billerica, MA). The blots were blocked in 5% non-fat dried milk in TBST for 1 h at room temperature, and incubated overnight with primary antibodies against p-Akt (1:2000), total Akt (1:2000, Epitomics), p-ERK1/2 (1:1000, abcam), total ERK1/2 (1:1000, abcam), p-p38 (1:300, abcam), total p38 (1:1000, abcam), p-mTOR (1:2000, abcam) and total mTOR (1:1000, abcam) at 4°C. After three washes with TBST, the blots were incubated with a horseradish peroxidase (HRP)-conjugated secondary antibody (Amersham Biosciences, Piscataway, NJ) and visualized via enzyme-linked chemiluminescence using the ECL kit (Chemicon, Temecula, CA). The blots were analyzed by Image J software with total Akt, ERK1/2, p38 or mTOR as control.

### Measurement of intracellular ROS and glutathione content

For the observation of intracellular ROS and glutathione content, the cells were loaded with 10 μM fluorescence probe 2,7-dichlorodihydrofluorescein diacetate, acetyl ester (DCHF-DA; Molecular Probes, Eugene, OR) for 30 min or 50 μM monochlorobimane (MCB, Sigma) for 15 min at 37°C. The fluorescence was observed and captured under the Nikon confocal microscopy (Nikon, Japan). For the quantification of intracellular ROS content, the cells stained with DCFH-DA were harvested and the fluorescence intensity was measured using flow cytometry.

### Measurement of intracellular calcium

The intracellular calcium in TKE2 cells was measured using the Ca^2+^-sensitive fluorescent dye Fluo-3 AM (Beyotime). Briefly, the cells were washed once with PBS, and were loaded with 2.5 μM Fluo-3 AM for 30 min at 37°C. The fluorescence was observed and captured under the Nikon confocal microscopy (Nikon).

### Total Antioxidant Capacity Measurement

Cellular total antioxidant capacity was measured according to the manufacturer’s instructions using Total Antioxidant Capacity Assay Kit with a Rapid ABTS method (Beyotime). In brief, the cells were collected, homogenized, and incubated with 2,2'-azino-bis (3-ethylbenzthiazoline-6-sulfonic acid (ABTS) for 6 min. The absorption was measured at 414 nm. The results were calibrated to the concentration of total protein in the samples.

### Mitochondria membrane potential staining

For the measurement of mitochondrial membrane potential, the cells were preloaded with 5 μg/ml 5,5’,6,6’-tetrachloro-1,1’,3,3’-tetraethyl-benzimidazole-carbocyanide iodine (JC-1; Beyotime) for 15 min. The fluorescence was observed and measured with the Nikon confocal microscopy.

### Statistical analysis

Data in this study were representative of more than three different experiments and presented as the means±SD. Statistical analysis was performed using SPSS 13.0 software (SPSS, Chicago, IL, USA) with one way ANOVA test. Differences were considered statistically significant at p<0.05.

## Results

### Hyperosmotic stress induces the apoptosis of corneal epithelial cells

**Fig 1:**
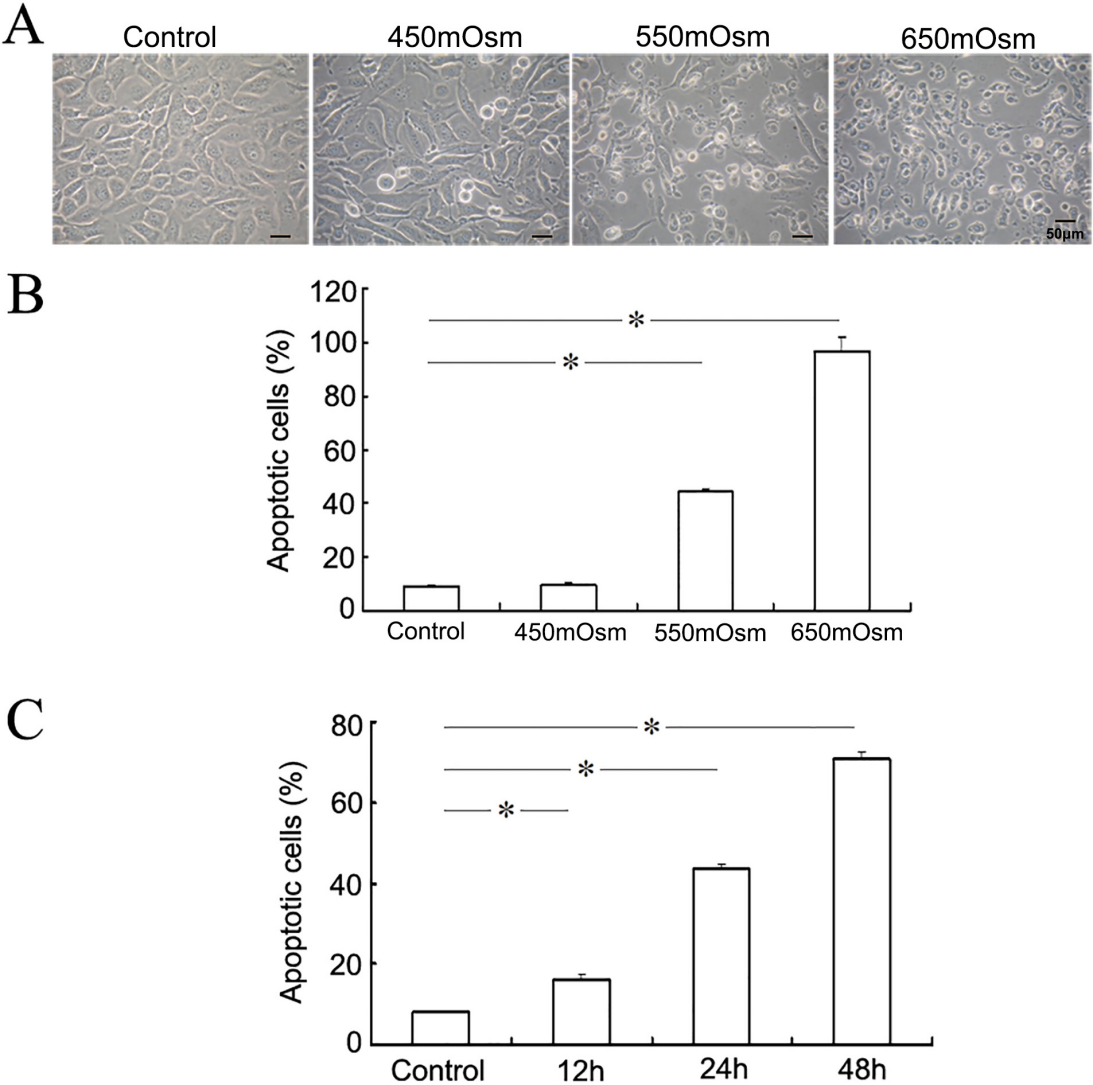
Hyperosmotic stress induces a dose- and time-dependent apoptosis in mouse corneal epithelial cells. Mouse corneal epithelial cells were treated with varying osmolarities (450, 550 or 650 mOsm) by addition of glucose for 24 h. The apoptotic cells were observed under inverted contrast microscopy (A) and detected by staining with FITC-Annexin V/PI and FACS analysis (B). Mouse corneal epithelial cells were treated with 550 mOsm hyperosmotic stress by addition of glucose for 12, 24 or 48h, and the apoptotic cells were investigated by staining with FITC-Annexin V/PI and FACS analysis (C). Hyperosmotic stress treatment induced the apoptosis of mouse corneal epithelial cells in a dose and time-dependent manner (B, C).

To investigate the effect of hyperosmotic stress on the survival of corneal epithelial cells, we treated mouse TKE2 cells in varying osmolarities (450, 550 or 650 mOsm) medium achieved by addition of glucose for 12, 24 or 48 h. The apoptotic cells were counted as the percent of Annexin V+/PI+ and Annexin V+/PI- cells followed by Annexin V/PI staining and FACS analysis. From the morphological observation, glucose treatment caused the shrunken and detachment of cultured corneal epithelial cells, especially when the osmolality was above 550 mOsm (Fig 1A). According to the results of FACS analysis, hyperosmolar conditions induced the apoptosis of mouse corneal epithelial cells via a dose- and time-dependent manner, with 45.23±2.45% apoptotic cells at 550 mOsm treatment for 24 h (Fig 1B and 1C). Thus the 550 mOsm hyperosmolar medium was used in most of the next studies.

### SP inhibits hyperosmotic stress-induced apoptosis of corneal epithelial cells

**Fig 2:**
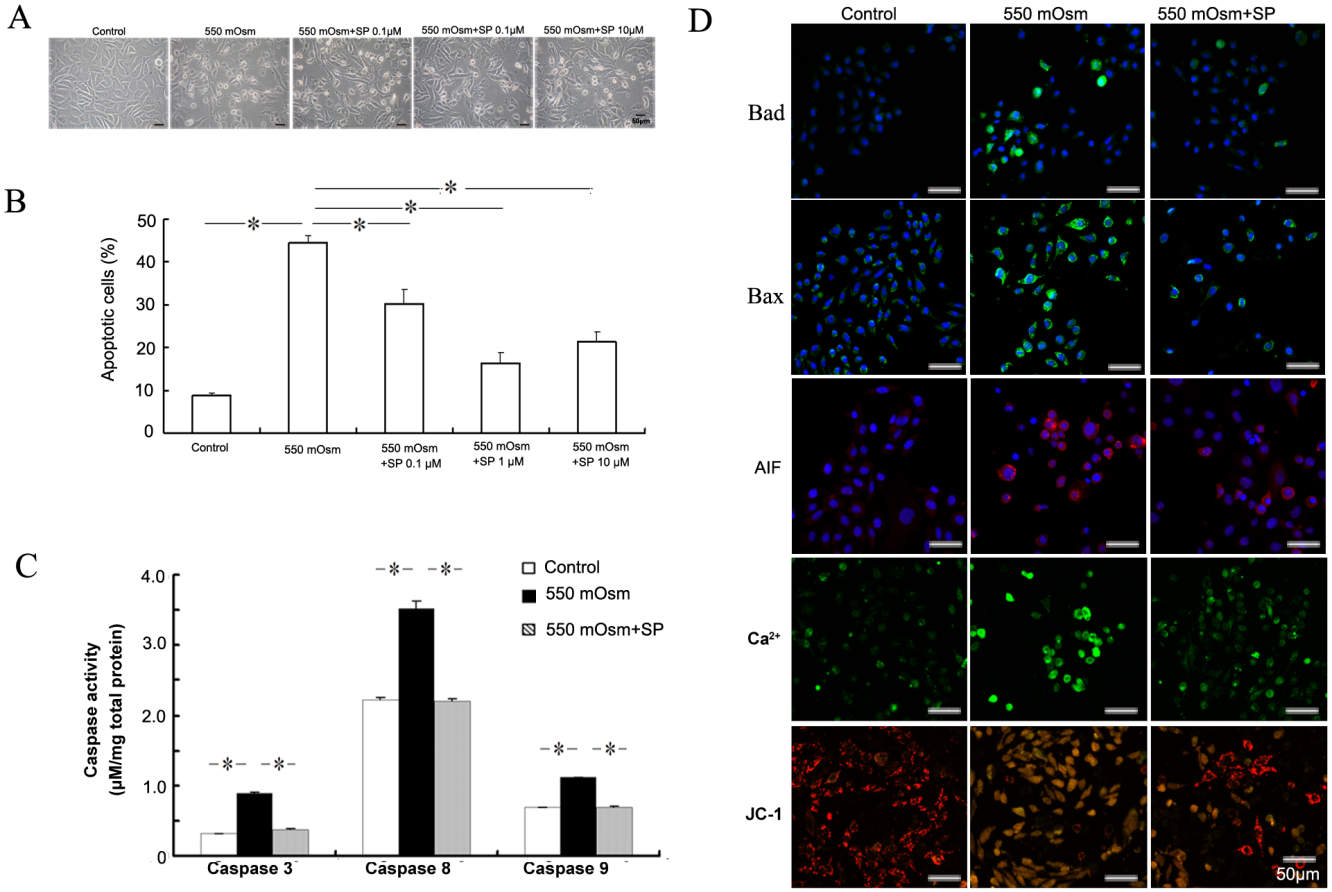
SP protects from hyperosmotic stress-induced apoptosis of corneal epithelial cells. Mouse corneal epithelial cells were treated with 550 mOsm hyperosmotic stress by addition of glucose with or without 0.1, 1 or 10 μM SP for 24 h. Cell morphology was observed under inverted contrast microscopy (A). The apoptosis was evaluated by FACS analysis followed by FITC-Annexin V/PI staining (B), caspase activity measurement (C), and the detection of Bcl-2-associated death promoter (Bad), BCL2-associated X protein (Bax), apoptosis inducing factor (AIF), Ca^2+^ and mitochondrial membrane potential (JC-1 staining) (D).

To assess the anti-apoptotic effect of SP in hyperosmolar medium treated corneal epithelial cells, mouse TKE2 cells were pre-treated with 0.1, 1 or 10 μM SP for 2 h before the addition of glucose. In comparison to glucose exposure, SP treatment protected the hyperosmotic stress-treated cells from the apoptotic morphological changes (Fig 2A). According to results of FACS analysis, the percent of annexin V+ apoptotic cells were reduced most significantly with the pre-treatment of 1 μM SP, as compared to that treated with 550 mOsm only (Fig 2B). So, the SP concentration of 1 μM was used in the following assays. Moreover, when compared to the control cells, the activities of caspase 3, 8, and 9 were increased approximately 2.76-, 1.58- and 1.62-fold with the cells cultured in hyperosmolar medium (550 mOsm), while the pretreatment with SP reversed the up-regulated caspase activities to the similar level as that of control cells (Fig 2C). In addition, hyperosmotic stress treatment also caused the increased fluorescence staining density of pro-apoptotic Bcl-2 proteins Bcl-2-associated death promoter (Bad) and BCL2-associated X protein (Bax), apoptosis inducing factor (AIF), Ca^2+^ and the changes of mitochondrial membrane potential (JC-1), while SP attenuated the increased staining density of Bad, Bax, AIF and Ca^2+^, and partially reversed the changes of mitochondrial membrane potential (Fig 2D). These results suggest that SP has an anti-apoptotic effect in corneal epithelial cells treated with 550 mOsm hyperosmotic stress, at least partially via the improvement of mitochondrial functions.

### SP prevents hyperosmotic stress-induced p-Akt reduction, ROS accumulation, and glutathione attenuation and total antioxidant capacity reduction

**Fig 3:**
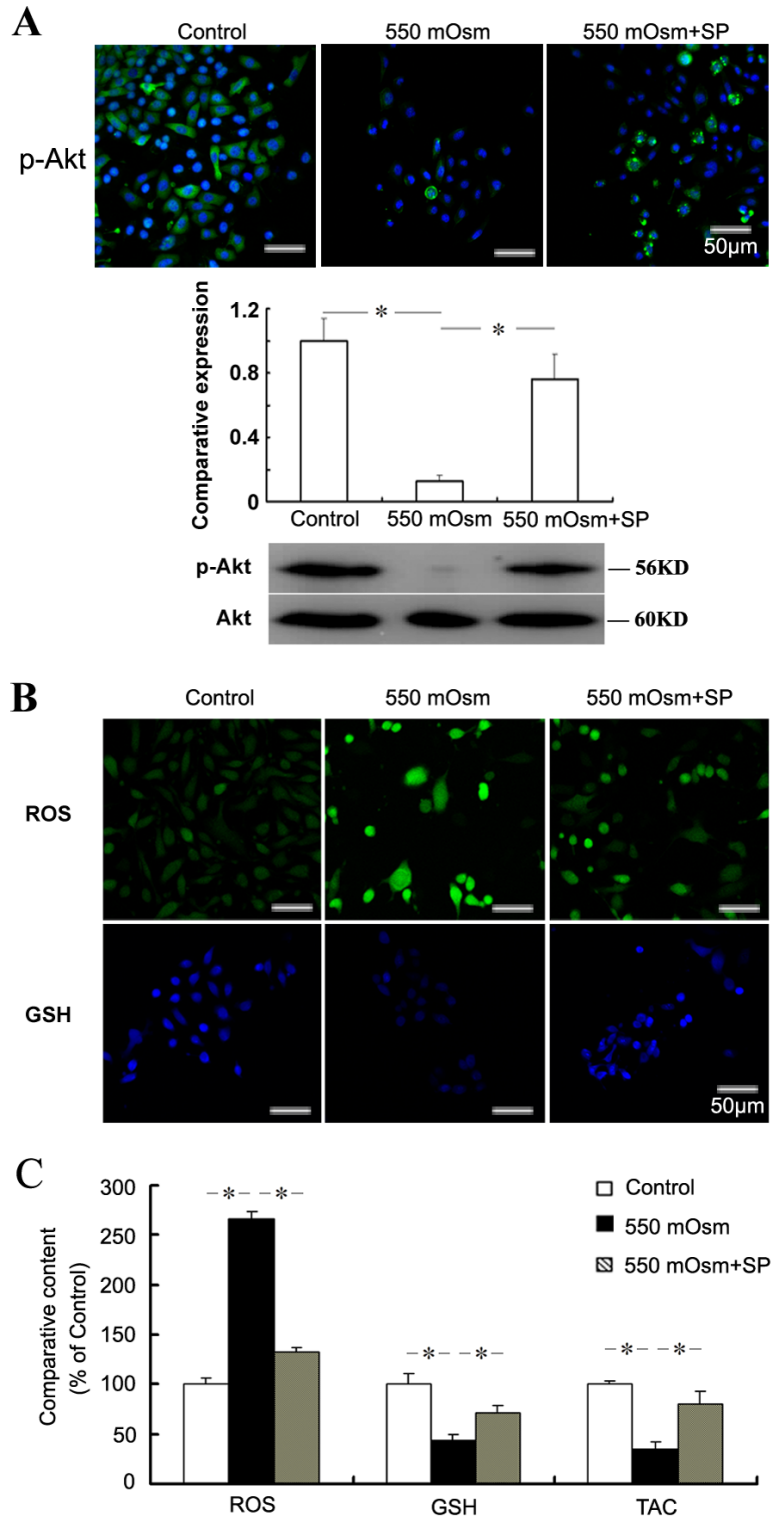
SP reactivates the phosphorylation of Akt and recovers the redox balance of corneal epithelial cells impaired by hyperosmotic stress. Mouse corneal epithelial cells were treated with 550 mOsm hyperosmotic stress by addition of glucose with or without 1 μM SP for 24 h. The phosphorylation of Akt was evaluated by Immunofluorescence staining or Western blot (A). The intracellular ROS and glutathione (GSH) levels were detected by staining with the fluorescence probes (B) and measured by the fluorescence intensity (C). The cellular total antioxidant capacity (TAC) was measured by the ABTS assay (C).

To investigate the protective mechanism of SP on the hyperosmotic stress-induced apoptosis, the cellular phosphorylated Akt level and redox balance were evaluated. From the results of western blot, we found that 550 mOsm hyperosmotic stress treatment induced an 87.27±2.14% reduction of the phosphorylated Akt level in corneal epithelial cells, which was restored to 76.15±1.54% of the normal p-Akt level when pre-incubated with SP (Fig 3A). More interestingly, when stained with the ROS and glutathione detector, we found that the treatment with 550 mOsm hyperosmotic stress alone caused the up-regulated ROS and down-regulated glutathione staining in comparison to the control cells in isotonic environment (310 mOsm), whereas the changes of staining density was partially reversed by the pre-treatment with SP. Representative staining of intracellular ROS and glutathione content was shown in Fig 3B. On the basis of biochemical analysis of cellular ROS and glutathione content, SP decreased intracellular ROS level by more than 2.01-fold, and increased the intracellular glutathione level by more than 1.64-fold compared with the cells treated with 550 mOsm hyperosmotic stress alone (Fig 3C). In addition, SP partially recovered the cellular total antioxidant capacity that impaired by the 550 mOsm hyperosmotic stress treatment based on measurement with ABTS assay (Fig 3C). The results suggest SP not only reactivated the Akt phosphorylation, but also recovered the redox balance of hyperosmotic stress-induced corneal epithelial cells.

### The reactivation of Akt partially mediates the anti-apoptotic effects of SP in hyperosmolar environment

**Fig 4:**
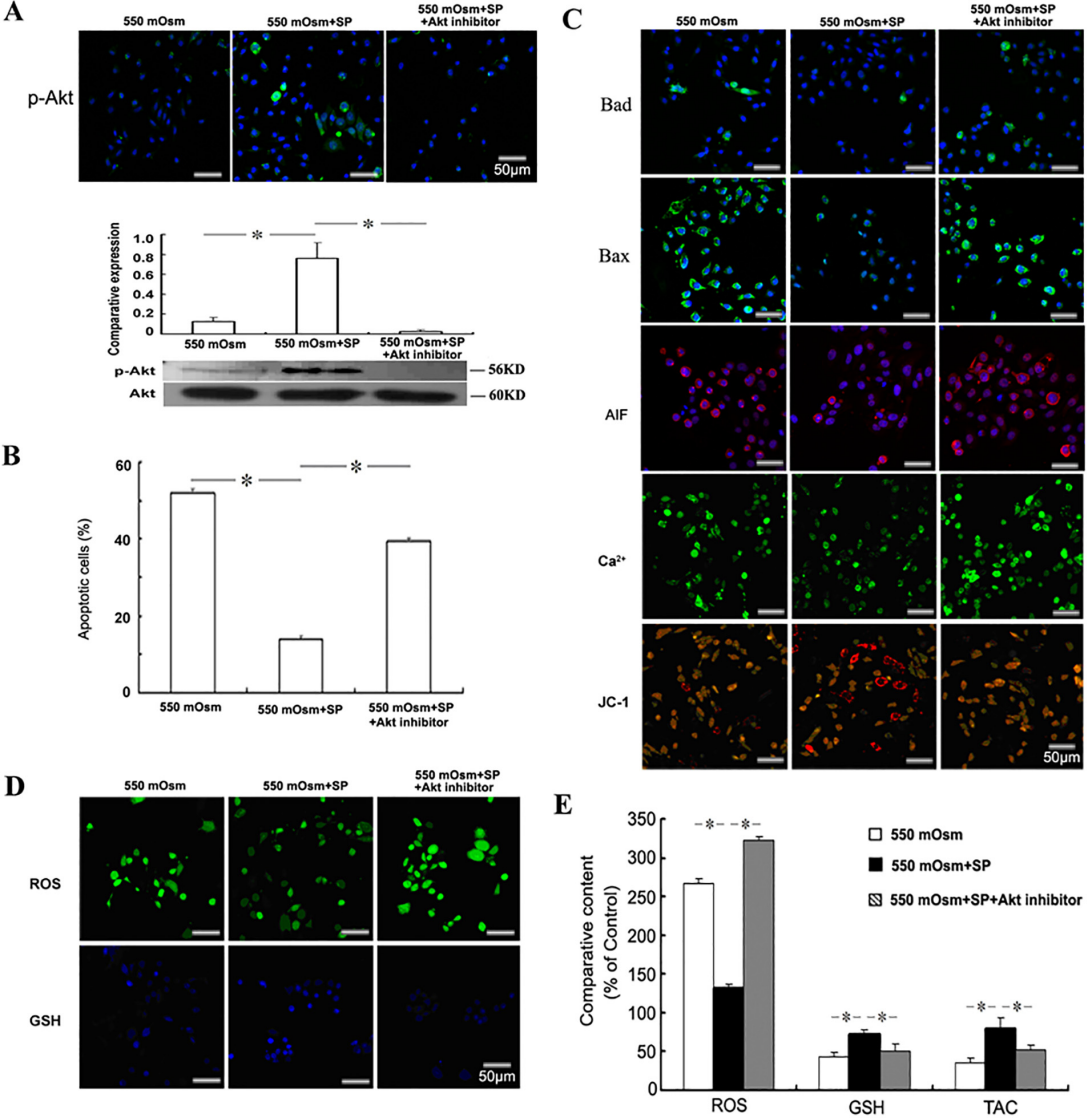
Role of Akt reactivation in the anti-apoptotic effects of SP. Mouse corneal epithelial cells were treated with 40 μM Akt inhibitor V and 1 μM SP 2 h before the addition of glucose for 24 h. The phosphorylation of Akt was evaluated by Immunofluorescence staining or Western blot (A). The apoptosis was evaluated by FACS analysis followed by FITC-Annexin V/PI staining (B), and the detection of Bad, Bax, AIF, Ca^2+^ and mitochondrial membrane potential (C). The intracellular ROS and glutathione (GSH) were detected by staining with the fluorescence probes (D) and measured by the fluorescence intensity (E). The cellular total antioxidant capacity (TAC) was measured by the ABTS assay (E).

To investigate if the Akt reactivation was involved in the anti-apoptotic effects of SP, mouse corneal epithelial cells were treated with the Akt inhibitor V and SP 2 h before the addition of glucose. Western blot showed that Akt inhibitor treatment inhibited completely the up-regulation of the p-Akt level induced by SP (Fig 4A). Moreover, the inhibition of Akt activation partially reversed the reduction of SP on the 550 mOsm hyperosmotic stress-induced apoptosis in corneal epithelial cells (Fig 4B), accompanied with the partial reversal of Bad expression, Bax expression, AIF production, intracellular Ca^2+^ accumulation and the loss of mitochondrial membrane potential (Fig 4C). In addition, we found that the Akt inhibition also reversed the improvement of intracellular ROS, glutathione content and the total antioxidant capacity by SP treatment in the hyperosmotic stress-treated corneal epithelial cells (Fig 4D and 4E). The results indicate that the reactivation of Akt partially mediated the anti-apoptotic effect of SP, while the inhibition of Akt reactivation inversely impaired the improvement of SP on the redox balance of hyperosmotic stress-treated corneal epithelial cells. Besides, AKT inhibitor V treatment alone on the expression of ROS, glutathione content and p-Akt were detected to exclude the possibility of Akt inhibition interfered the hyperosmolar environment (S1 Fig).

### The regulation of redox balance partially mediates the anti-apoptotic effects of SP in hyperosmolar environment

**Fig 5:**
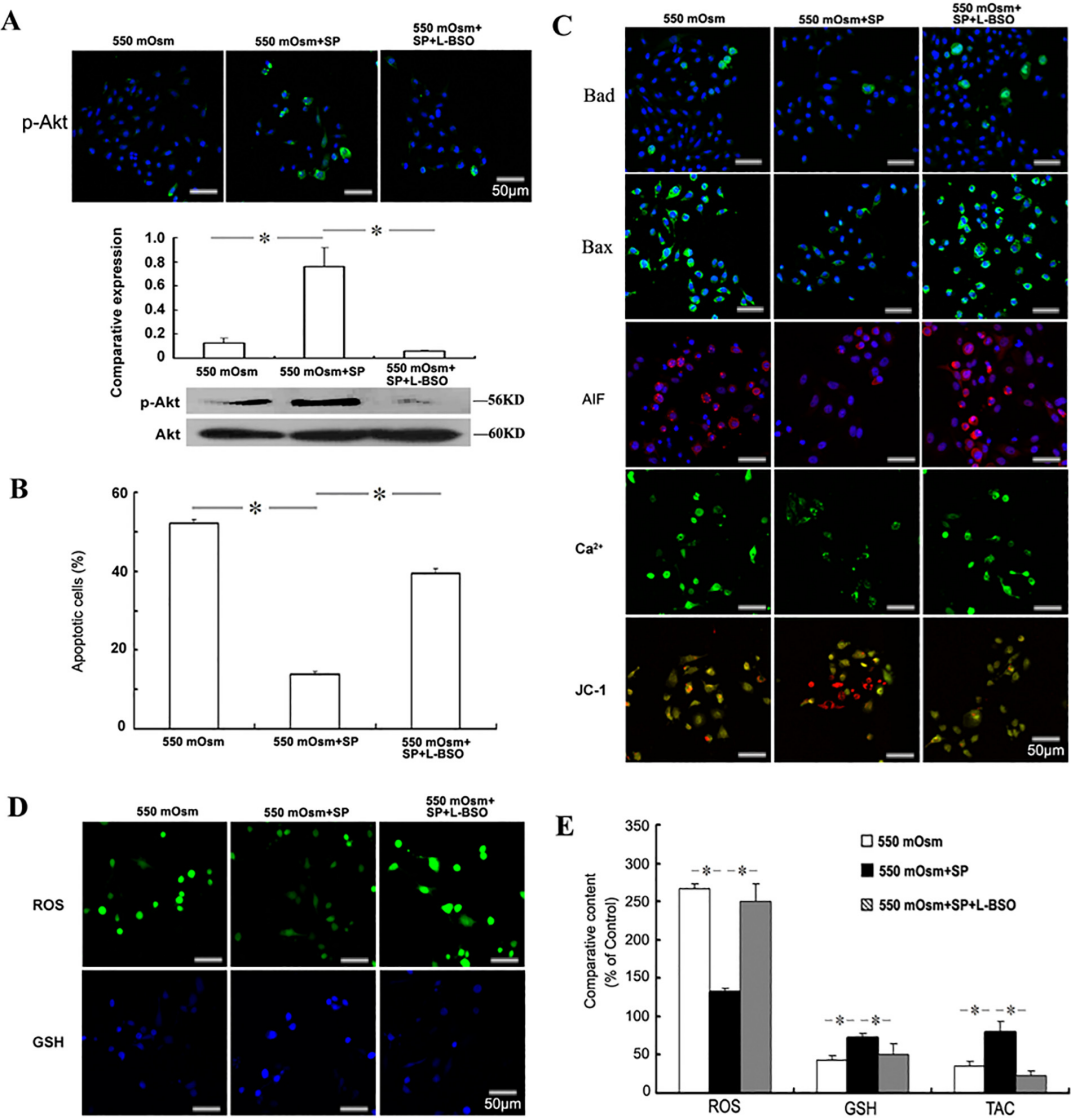
Role of redox regulation in the anti-apoptotic effects of SP. Mouse corneal epithelial cells were treated with 100 μM L-BSO and 1 μM SP 2 h before the addition of glucose for 24 h. The phosphorylation of Akt was evaluated by Immunofluorescence staining or Western blot (A). The apoptosis was evaluated by FACS analysis followed by FITC-Annexin V/PI staining (B), and the detection of Bad, Bax, AIF, Ca^2+^ and mitochondrial membrane potential (C). The intracellular ROS and glutathione (GSH) were detected by staining with the fluorescence probes (D) and measured by the fluorescence intensity (E). The cellular total antioxidant capacity (TAC) was measured by the ABTS assay (E).

To further determine whether the function of increase intracellular glutathione reduction induced by hyperosmotic stress exerts an important role in the anti-apoptotic effects of SP, L-BSO was used to induce intracellular glutathione deficiency. As shown in Fig 5A, L-BSO inhibited totally the increased level of p-Akt induced by SP. And glutathione scavenged by L-BSO partially reversed the reduction of SP on the 550 mOsm hyperosmotic stress (achieved by addition of glucose)-induced apoptosis (Fig 5B), Bad expression, Bax expression, AIF production, the intracellular Ca^2+^ accumulation and the loss of mitochondrial membrane potential (Fig 5C) in corneal epithelial cells. And as expected, L-BSO significantly reversed the improvement of intracellular ROS, glutathione content and the total antioxidant capacity by SP treatment in corneal epithelial cells cultured in 550 mOsm hyperosmolar medium (Fig 5D and 5E). These findings suggest that SP exerts the anti-apoptotic function partially by an oxidant scavenging-dependent mechanism. In addition, L-BSO treatment alone on the expression of ROS, glutathione content and p-Akt were examined to exclude the influence of glutathione depleting on the hyperosmolar environment (S1 Fig).

### The NK-1 receptor mediates the anti-apoptotic effects of SP in hyperosmolar environment

**Fig 6:**
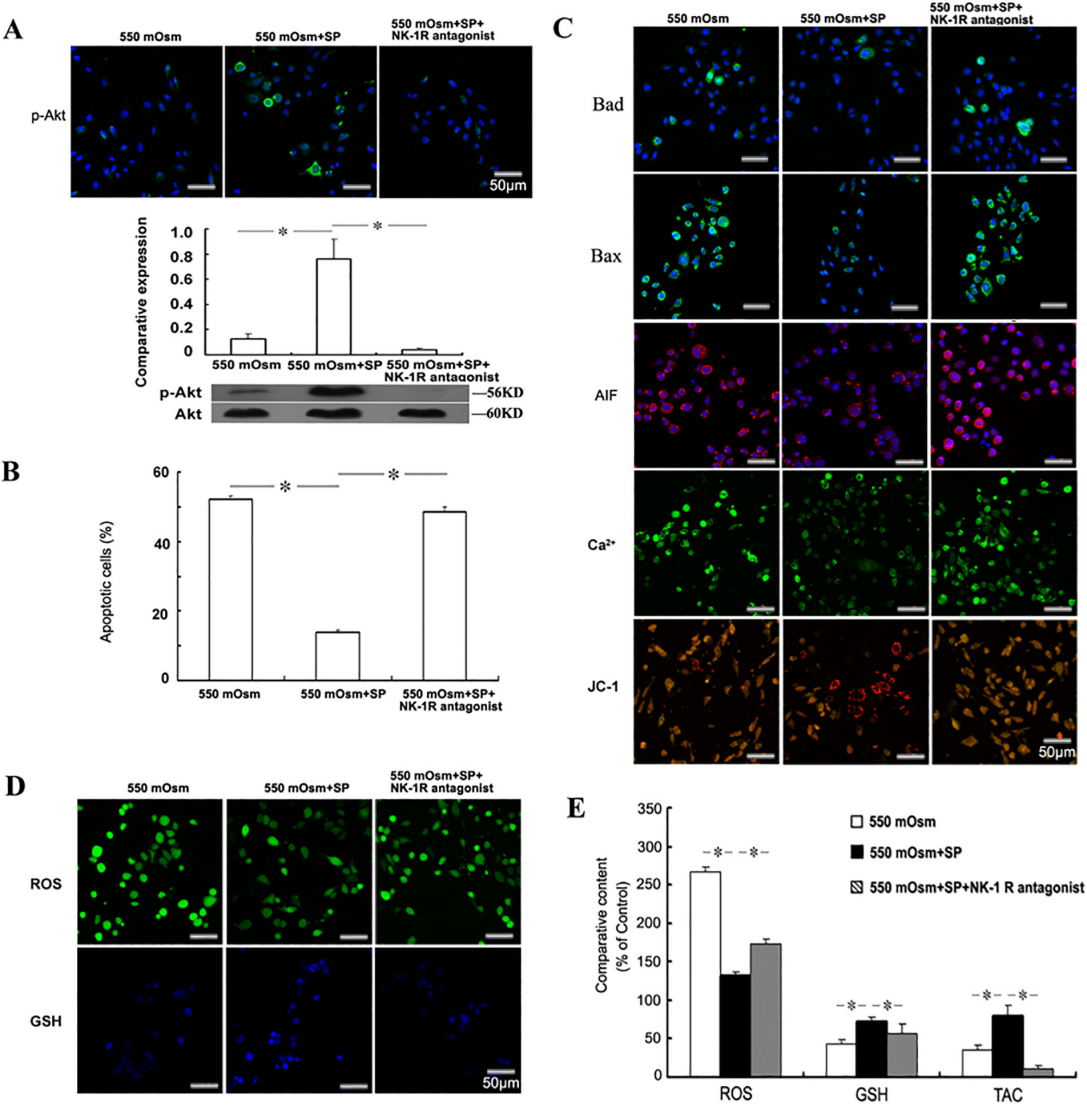
Role of NK-1 receptor in the anti-apoptotic effects of SP. Mouse corneal epithelial cells were treated with 1 μM NK-1 receptor antagonist L-733,060 with 1 μM SP 2 h before the addition of glucose for 24 h. The phosphorylation of Akt was evaluated by Immunofluorescence staining or Western blot (A). The apoptosis was evaluated by FACS analysis followed by FITC-Annexin V/PI staining (B), and the detection of Bad, Bax, AIF, Ca^2+^ and mitochondrial membrane potential (C). The intracellular ROS and glutathione (GSH) were detected by staining with the fluorescence probes (D) and measured by the fluorescence intensity (E). The cellular total antioxidant capacity (TAC) was measured by the ABTS assay (E).

To assess if the NK-1 receptor mediates the anti-apoptotic effects of SP in hyperosmolar environment, the NK-1 receptor specific antagonist L-733,060 (1 μM) was added with SP 2 h before the treatment of glucose. Western blot showed that the addition of NK-1 receptor antagonist fully reversed the Akt reactivation induced by SP and even become lower than that of 550 mOsm-treated cells (Fig 6A). As shown in Fig 6B, the NK-1 receptor antagonist almost completely blocked the reduction of apoptotic cell percent induced by SP in 550 mOsm hyperosmotic stress-treated corneal epithelial cells (48.41+1.56 apoptotic cells in the group of 550 mOsm+SP+NK-1 receptor antagonist treatment; 52.19+0.85 apoptotic cells in the group of 550 mOsm treatment alone), accompanied with the partial reversal of Bad expression, Bax expression, AIF production, intracellular Ca^2+^ accumulation and the loss of mitochondrial membrane potential (Fig 6C). Moreover, the regulatory effects of SP on cellular ROS, glutathione content and the total antioxidant capacity were also dramatically reversed by NK-1 receptor antagonist (Fig 6D and 6E). The results suggest that the NK-1 receptor mediates the anti-apoptotic effects of SP in hyperosmotic stress-treated corneal epithelial cells through the regulation of Akt activation and redox balance. In addition, the expression of major intracellular free radical scavengers in TKE2 cells, including MnSOD, catalase, NQO1 and Hmox1 were detected immunofluorescent staining (S2 Fig). The results revealed that 550 mOsm hyperosmotic stress treatment inhibited the expression of MnSOD, catalase, NQO1 and Hmox1, which can be recovered by SP application. However NK-1 receptor antagonist (L-733,060) surpressed the up-regulation of MnSOD, catalase, NQO1 and Hmox1 level induced by SP. In addition, L-733060 treatment alone on the expression of ROS, glutathione content and p-Akt were examined to exclude the interference of the NK-1 receptor antagonist on the hyperosmolar environment (S1 Fig).

## Discussion

In this study, we found firstly that the neuropeptide substance P inhibited the hyperosmotic stress-induced apoptosis of mouse corneal epithelial cells. Moreover, SP promoted the recovery of phosphorylated Akt level, mitochondrial membrane potential, Ca^2+^ contents, intracellular reactive oxygen species (ROS) and glutathione levels that impaired by hyperosmotic stress. However, the antiapoptotic capacity of SP was partially suppressed by Akt inhibitor or glutathione depleting agent, while the neurokinin-1 (NK-1) receptor antagonist impaired Akt activation and ROS scavenging that promoted by SP addition. In conclusion, SP protects corneal epithelial cells from hyperosmotic stress-induced apoptosis through the mechanism of Akt activation and ROS scavenging via the NK-1 receptor.

The corneal epithelial cells act as a barrier to protect the cornea, apoptosis of ocular surface epithelial has been involved in the pathogenesis of the ocular disease that develops in hyperosmotic [35, 38, 40, 41] environments. In this study, we proposed an experimental model in vitro in which corneal epithelial cells (TKE2 cells) were exposed to hyperosmolar medium achieved by addition of glucose to induced acute apoptosis. Similar as the research of Garrett Q [40], our results show that the osmolarity of 550 mOsm and an exposure time of 24 h were optimal to study the responses of hyperosmotic stress-induced apoptosis.

It has been reported widely that SP has anti-apoptotic ability in many type of cells [1, 4–8]. Now we further demonstrate that SP inhibits hyperosmotic stress-induced apoptosis in cultured corneal epithelial cells by detection the well known apoptotic markers of annexin V, caspase 3/8/9, Bad, Bax and AIF. In accordance with previous studies in colonocytes and tenocytes [7, 11], our findings also show that SP stimulates phosphorylation of Akt which is known as an anti-apoptotic protein kinase. When treated with Akt inhibitor V, the anti-apoptotic effect of SP was significantly suppressed, indicating that Akt-related signaling is important for the inhibition effect of SP on hyperosmotic stress-induced apoptosis. However, the study of how SP regulates apoptosis through Akt signaling is still inadequate.

Previous reports have shown excessive presence of cellular ROS affect mitochondrial membrane potential, increase cytoplasm Ca^2+^ and eventually cause apoptosis [42–44]. Our previous study has confirmed the attenuating function of SP in the hyperglycemia-induced oxidative stress of corneal epithelium [45]. In accordance with the previous study, our results in the present study reveal that in this experimental conditions SP inhibited hyperosmotic stress-induced ROS and Ca^2+^ accumulation, GSH reduction, the decrease of cellular total antioxidant capacity and the impairment of mitochondrial membrane potential in corneal epithelial cells. However, when the intracellular glutathione deficiency of corneal epithelial cells was induced by L-BSO, the anti-apoptotic effect of SP was decreased. And treatment with L-BSO markedly inhibited the reactivated fuction of SP on phosphorylation of Akt. Studies have shown the overproduction of intracellular ROS levels decreases the phosphorylation of Akt [46]. Moreover, treatment with the Akt inhibitor V significantly prevented the modulation effects of SP on oxidative stress. In this study, our findings show dephosphorylation of Akt stimulates ROS generation, suggesting ROS and Akt signaling may have interactive relation.

It is known that the biological actions of SP are mainly mediated by NK-1 receptor [45–47]. Previous study shows that SP reduces TNF-α-induced apoptosis of human tenocytes through stimulation of the NK-1 receptor [47]. Also we demonstrated the potential relationship between SP and its receptor NK-1 in modulating hyperosmotic stress-induced apoptosis in TKE2 cells. We blocked NK-1 receptor in SP-treated TKE2 cells by the use of non-peptide NK-1 receptor antagonists L-733,060, a piperidine derivative showing high affinity for the NK-1 receptor in vitro. Inhibition of SP-NK-1 receptor signaling by L-733,060 caused increased apoptosis events, decreased phosphorylation of Akt level, increased ROS and Ca^2+^ generation, reduction of GSH level and cellular total antioxidant capacity, and more impaired mitochondrial membrane potential compared with SP treated alone. All the data mentioned above suggest that the biological functions of SP were blocked markedly by treatment with NK-1 receptor antagonist, confirming that the effect of SP is mediated via a NK-1 receptor specific pathway. The effect of SP-NK-1 receptor signaling on phosphorylation of Akt in the current work is in concord with previous studies [7, 11]. However, to our knowledge, this is the first report suggests that SP could decrease ROS accumulation through NK-1 receptor specific pathway in a cell apoptosis system.

In addition, we also examined the role of SP on some other signaling pathway involved in hyperosmotic stress-induced apoptosis including ERK1/2, p38 and mTOR [48, 49]. We found that 550 mOsm hyperosmotic stress (achieved by addition of glucose) treatment induced a reduction of p-ERK1/2 and p-mTOR in TKE2 cells, which were partially restored by the pre-treatment with SP. And Nk-1R antagonist, Akt inhibitor V or L-BSO treatment inhibited completely the up-regulation of the p-ERK1/2 level induced by SP. In addition, Nk-1R antagonist and L-BSO treatment inhibited completely the up-regulation of the p-mTOR level induced by SP. But Akt inhibitor V didn’t suppress the up-regulation of the p-mTOR level induced by SP. However, hyperosmotic stress or SP didn’t change the p-p38 level significantly (S3 Fig). These finding suggested that several signaling pathways maybe involved the regulation of SP on hyperosmotic stress-induced apoptosis, which should be further investigated in the future study.

## Conclusions

In the present study, we provide the first sample of the anti-apoptotic capacity of substance P not only through the activation of Akt signaling, but also through the promotion of ROS scavenging via NK-1 receptor.

## Supporting Information

S1 Fig Role of different inhibitors treated alone on the expression of ROS, GSH, and p-Akt. The expression of ROS, GSH, and p-Akt in TKE2 cells treated with different inhibitors (Akt inhibitor V, L-BSO and NK-1R antagonist) alone with or without 550 mOsm hyperosmotic stress treatment were detected using immufluorescent staining. The results showed that different inhibitors didn’t influence the hyperosmolar environment obviously.
(TIF)

S2 Fig SP attenuates hyperosmotic stress-induced reduction of intracellular free radical scavengers in corneal epithelial cells. Immunoflurorescent staining showed that the protein levels of catalase, Hmox1, NQO1, and MnSOD were decreased in hyperosmotic environment, which can be re-increased by SP application. However NK-1 receptor antagonist surpressed the promotion of catalase, Hmox1, NQO1, and MnSOD induced by SP.
(TIF)

S3 Fig Role of SP on the regulation of ERK1/2, p38 and mTOR signals. The signaling of ERK1/2, p38 and mTOR were determined using western blot, the results showed that 550 mOsm hyperosmotic stress (achieved by addition of glucose) treatment induced a reduction of p-ERK1/2 and p-mTOR in TKE2 cells, which were partially restored by SP application. Nk-1R antagonist, Akt inhibitor V or L-BSO treatment inhibited completely the up-regulation of the p-ERK1/2 level induced by SP. In addition, Nk-1R antagonist and L-BSO treatment inhibited completely the up-regulation of the p-mTOR level induced by SP. But Akt inhibitor V didn’t suppress the up-regulation of the p-mTOR level induced by SP. However, hyperosmotic stress or SP didn’t change the p-p38 level significantly.
(TIF)
