# Supplementary figures and images for: Substance P Inhibits Hyperosmotic Stress-Induced Apoptosis in Corneal Epithelial Cells through the Mechanism of Akt Activation and Reactive Oxygen Species Scavenging via the Neurokinin-1 Receptor
Source: PLoS One. 2016 Feb 22;11(2):e0149865. doi: 10.1371/journal.pone.0149865 (PMC4762577; doi:10.1371/journal.pone.0149865)

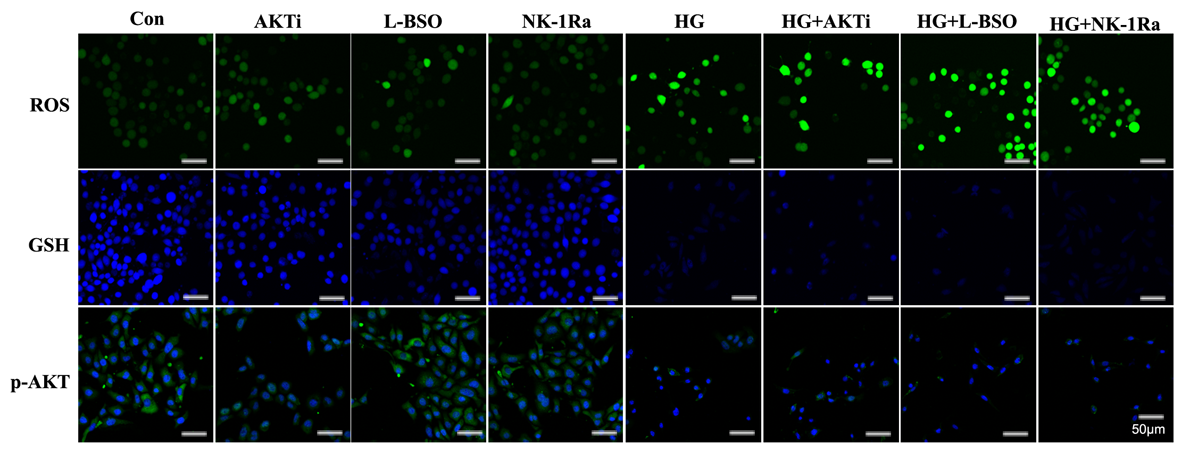

Supplement: S1 Fig — The expression of ROS, GSH, and p-Akt in TKE2 cells treated with different inhibitors (Akt inhibitor V, L-BSO and NK-1R antagonist) alone with or without 550 mOsm hyperosmotic stress treatment were detected using immufluorescent staining. The results showed that different inhibitors didn’t influence the hyperosmolar environment obviously. (TIF) [file pone.0149865.s001.tif]

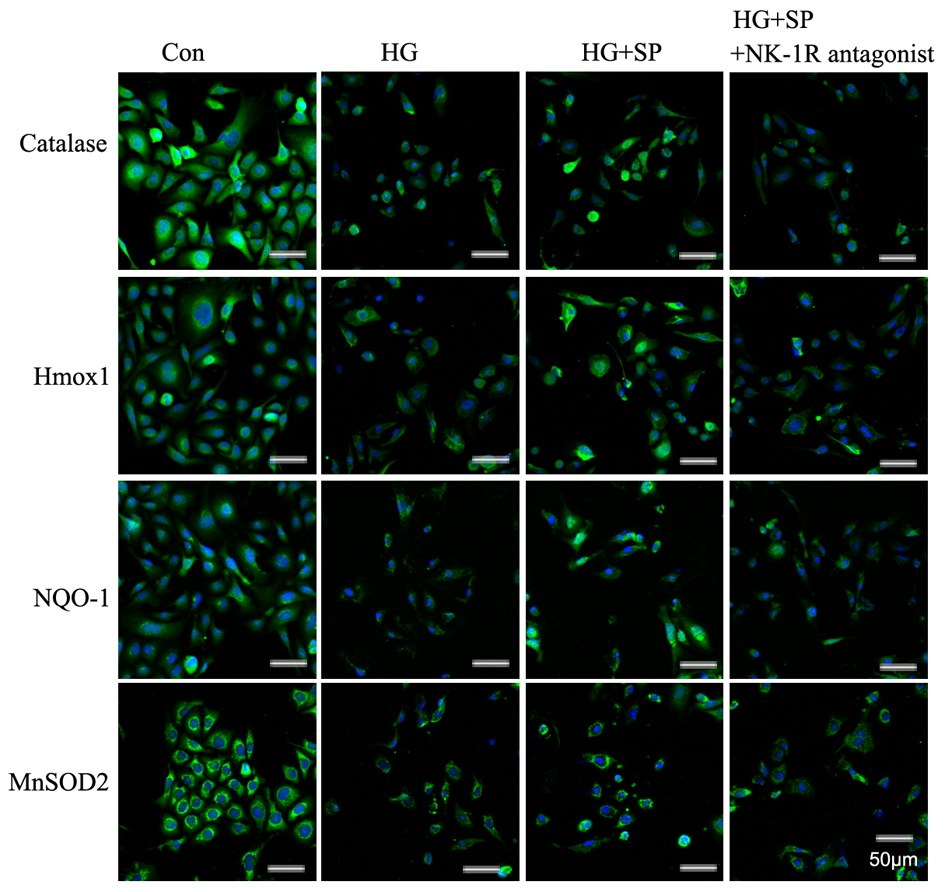

Supplement: S2 Fig — Immunoflurorescent staining showed that the protein levels of catalase, Hmox1, NQO1, and MnSOD were decreased in hyperosmotic environment, which can be re-increased by SP application. However NK-1 receptor antagonist surpressed the promotion of catalase, Hmox1, NQO1, and MnSOD induced by SP. (TIF) [file pone.0149865.s002.tif]

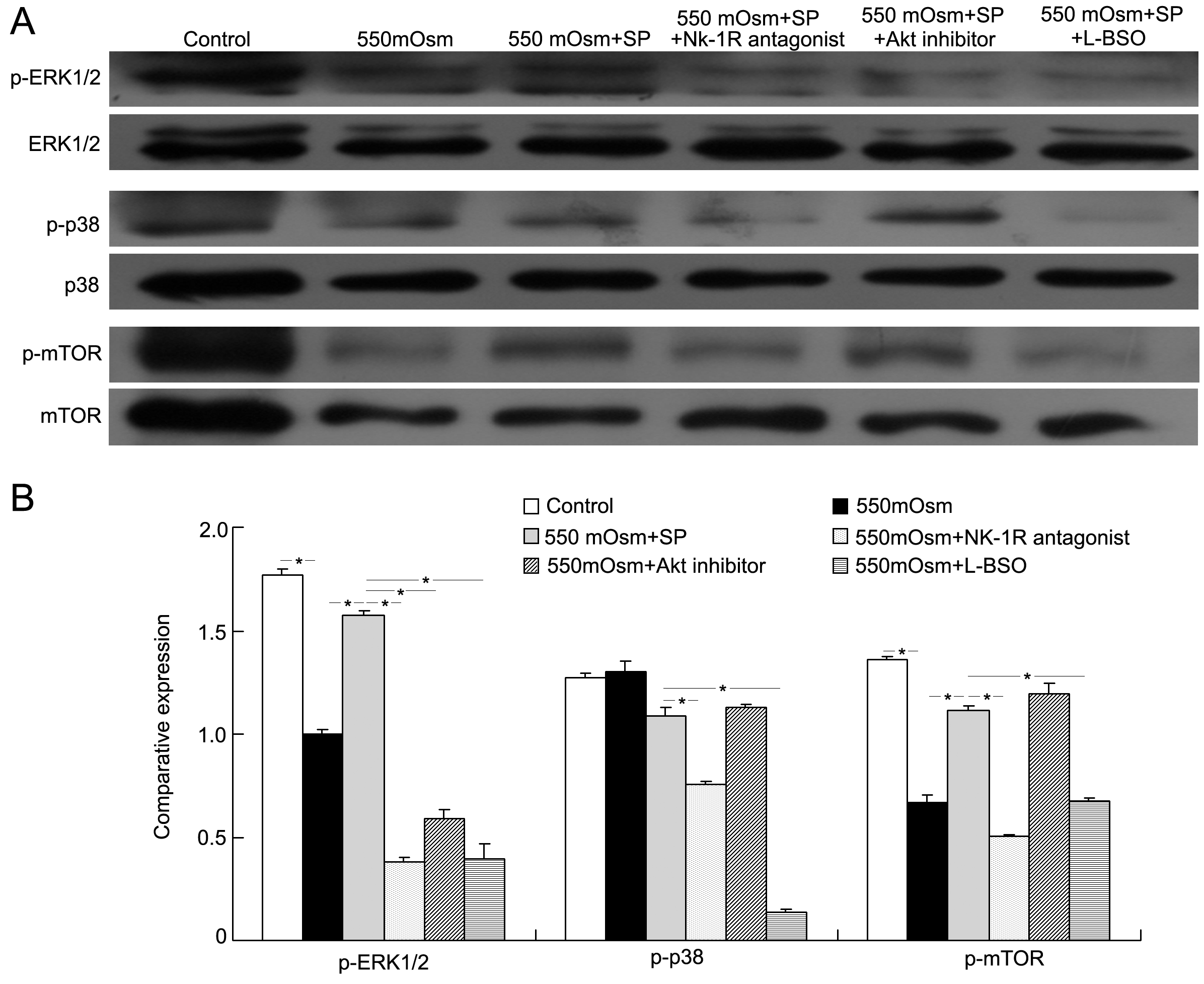

Supplement: S3 Fig — The signaling of ERK1/2, p38 and mTOR were determined using western blot, the results showed that 550 mOsm hyperosmotic stress (achieved by addition of glucose) treatment induced a reduction of p-ERK1/2 and p-mTOR in TKE2 cells, which were partially restored by SP application. Nk-1R antagonist, Akt inhibitor V or L-BSO treatment inhibited completely the up-regulation of the p-ERK1/2 level induced by SP. In addition, Nk-1R antagonist and L-BSO treatment inhibited completely the up-regulation of the p-mTOR level induced by SP. But Akt inhibitor V didn’t suppress the up-regulation of the p-mTOR level induced by SP. However, hyperosmotic stress or SP didn’t change the p-p38 level significantly. (TIF) [file pone.0149865.s003.tif]
